# The Simulation-Based Approach for Random Speckle Pattern Representation in Synthetically Generated Video Sequences of Dynamic Phenomena

**DOI:** 10.3390/s22239489

**Published:** 2022-12-05

**Authors:** Paweł Zdziebko, Ziemowit Dworakowski, Krzysztof Holak

**Affiliations:** Department of Robotics and Mechatronics, AGH University of Science and Technology, al. Mickiewicza 30, 30-059 Krakow, Poland

**Keywords:** finite element analysis, vision systems, rendering, random speckle patterns, Blender

## Abstract

Structural health monitoring systems that employ vision data are under constant development. Generating synthetic vision data is an actual issue. It allows, for example, for obtention of additional data for machine learning techniques or predicting the result of observations using a vision system with a reduced number of experiments. A random speckle pattern (RSP) fixed on the surface of the observed structure is usually used in measurements. The determination of displacements of its areas using digital image correlation (DIC) methods allows for extracting the structure’s deformation in both static and dynamic cases. An RSP modeling methodology for synthetic image generation is developed within this paper. The proposed approach combines the finite element modeling technique and simulation results with the Blender graphics environment to generate video sequences of the mechanical structure with deformable RSP attached to it. The comparative analysis showed high compliance of the displacement between the synthetic images processed with the DIC method and numerical data.

## 1. Introduction

Image processing algorithms and computer vision systems have become essential tools for inspecting and monitoring structures [1] in the fields of mechanical [2], aviation [3], and civil engineering applications [4,5]. Image data contains full-field displacement and deformations of objects of interest. Moreover, a series of images taken at consecutive time moments encodes information about the change in the state of the structure. If one incorporates a high-speed camera into the vision-based monitoring system, dynamic phenomena can be recorded as well. Structural deformation is often obtained using digital image correlation (DIC) [6]. In this technique, it is necessary to prepare a test subject before the measurement is carried out. A high-contrast random speckle pattern (RSP) is placed on its surface. This method is mainly used for vision-based strain analysis of material specimens subjected to various mechanical testing procedures. However, it has been successfully carried out for the measurement of large structure displacement [7,8]. 

Developing vision-based systems for such applications depends upon large image and video databases. It often requires time-consuming preparation steps, including placing markers or RSP on measured surfaces, positioning the camera system with respect to the object, choosing the focal length and measurement distance, and obtaining favorable lightning conditions, if possible. For the correct development of image processing algorithms for structural state evaluation or damage detection, it is necessary to take images of the object in various conditions. In the case of laboratory setups, this is time-consuming and costly. Moreover, it may be impossible in the case of real-life engineering structures since damaged structures are not common. Therefore, there is a need for new numerical tools which make it possible to perform computational simulations for measurement vision systems. 

A lot of progress has been made in the fields of computer-aided design, computer graphics, and virtual and augmented reality. Computer graphics software has allowed the creation of photo-realistic images and videos. It helps researchers generate large amounts of data, including different geometries of structures seen from numerous viewpoints, textures and colors of surfaces, and lighting conditions. The use of synthetically generated data for the development of image analysis methods has been reported in the literature. One of the recent applications was in the generation of synthetic view datasets to train a deep neural network for object classification [9]. This approach increased the size of datasets and introduced new imaging conditions that were absent in the original images. The first approach uses game engines to render images in a shorter time, and the second one applies physics-based ray tracing algorithms to produce photo-realistic images with complex lighting effects. The authors [5] presented a method of physics-based modeling of structures with different damage conditions for synthetic image data generation. In the proposed approach, a deep neural network has been used to identify changes occurring in the miter gate. The network was trained by data generated by computer graphics software. The training set included various damage conditions such as cracks and corrosion under variable lighting conditions. Synthetic image generation has found application in developing robots and autonomous vehicles [10] and semantic segmentation of urban scenes [11]. It has been proven that this approach improves the performance of self-driving cars in natural urban environments [12,13]. Researchers have begun using 3D synthetic data in vision-based monitoring systems. Hoskere et al. [14] presented physics-based damage models incorporated into structural damage inspection systems and Narazaki et al. [15,16] developed physics-based models for displacement and strain measurements. In [17], the synthetic image generation approach and deep learning method have been applied in post-earthquake inspection of reinforced concrete viaducts.

Our team recently published a numerical environment for synthetic image generation [18]. The simulations aimed to create realistic-looking scenes (images) using computer resources. The proposed approach allows for the simulation of deformations of mechanical structures captured by the vision system, considering all necessary properties of the system. Structural deformations were determined using the Finite Element Analysis (FEA). At a later stage, the Blender graphics program allowed generation of realistic-looking renders that presented the studied mechanical structures based on FEA results. Properties of the modeled vision system in the Blender program are taken into account, including the camera’s position relative to the object, the focal length, the sensor size, and the scene’s lighting. As a result, images of simulated ‘virtual experiments’ are rendered using a graphics processing unit. Generated images can be further utilized as synthetic vision data for testing image processing algorithms or used as a guideline to determine the optimal vision system’s settings during observation in a real-life experiment conducted later. It is worth noting that while RSP is a crucial tool in DIC-based image and video interpretation, no attempts at a viable simulation of deformable RSP have been made up to this date.

In this paper, we provide an extension of the previous work [18], including two significant contributions: 

1. Development of the simulation approach to cover dynamic phenomena and the generation of video sequences as the output vision data;

2. A novel framework for simulation of deformable RSP behavior based on the FE model.

There are a few papers focusing on rendering the deformed RSP such as those introduced by Sur et al. [19,20], but none of them use the FEM-based modeling technique to assess the dynamic response of the structure with RSP attached to it, as is done in our approach. 

The paper is organized as follows: Section 2 presents the methodology for random speckle pattern representation. In Section 3, the developed version of the FEM plus Blender simulation algorithm is discussed. Subsequently, Section 4 presents the case study results; finally, Section 5 summarizes the work.

## 2. Materials and Methods

### 2.1. Random Speckle Pattern

**Figure 1:**
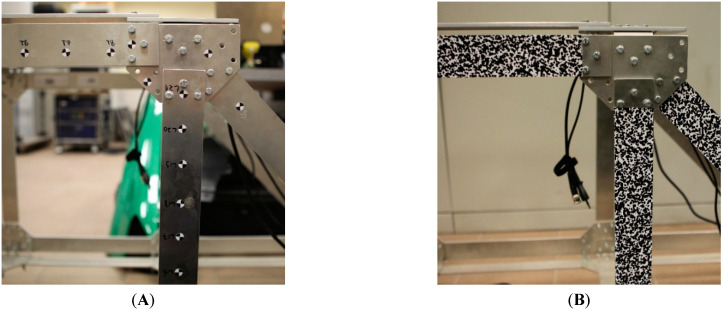
Structure prepared for vision measurements using vision markers (**A**) and RSP (**B**).

The Digital Image Correlation (DIC) is a common non-contact strain and deformation measurement method applied mainly in the mechanical testing of materials. Usually, in the first step, the surface of the specimen is coated with a random speckle pattern (RSP) of high contrast to improve the accuracy of the measurement. The image of an unloaded specimen is taken as a reference. It is divided into a number of rectangular patches, forming a grid. The deformation of the object under successively applied loads is recorded by a camera. In order to compute each image patch’s displacement course with respect to its position in the reference image, it is matched with each frame in the sequence by the maximization of some similarity criterion. Due to its robustness to uniform changes in the brightness, the normalized cross-correlation (NCC) coefficient is chosen as the criterion. A dense displacement field may be calculated if all patches of a grid are tracked in all frames of the sequence. It can be used to compute various components of the strain tensor. If one applies a stereovision DIC system, it is possible to compute 3D displacements and 3D strains as well. RSP (also called Digital Speckle Pattern) is an excellent alternative to vision markers (see Figure 1). RSP involves high local contrast and low repeatability of the pattern to ensure reliable and high accuracy tracking of points of interest while using the DIC methods. Using RSO allows for picking points of interest using a very dense (possibly subpixel interpolation) grid. In practical scenarios, RSP is sprayed on the surface using black and white paint or attached with stickers with printed patterns.

**Figure 2:**
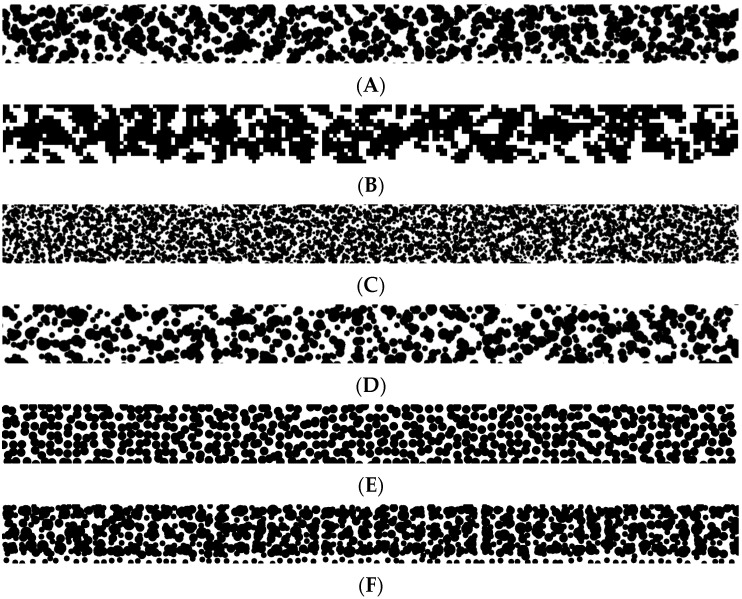
Examples of various RSPs: (**A**) RSP chosen as a source to be modeled in this work, (**B**) rescaled RSP for the purpose of FE model used in this work, (**C**) example of RSP generated for a smaller field of view, (**D**) example of RSP with higher sparsity, (**E**) example of RSP optimized for a particular scale, and (**F**) an example of RSP with lower influence of random speckle displacements.

Various pattern templates are recognized in the literature, some of which are presented in Figure 2. While there are different approaches to designing RSPs, in general, there are two major categories of RSPs used: monoscale and multiscale. The former is designed to work with a particular image scale, that is, a particular distance from the vision sensor to the object. As reported by Chen et al., in such a situation, the speckle size should have a diameter of roughly 4 pixels [21]. The latter is designed to work at different scales, meaning that the structure can be placed at various distances from the camera and still be measured with acceptable accuracy and they contain either speckles of different sizes or rely on the speckles forming higher scale structures.

Modeling RSP or vision marker behavior in images rendered from FEA results is challenging because markers and RSPs need to be attached to the modeled structure before starting the dynamic simulation and not just painted over the already-bent model. To this end, marker locations should correspond to particular nodes of an FE model. When it comes to RSP, however, due to its local uniqueness, it is required that the full RSP be coded into the FE model, with each pixel of RSP corresponding to the particular FE of the model.

In this work, we are using the authors’ Matlab script for RSP generation based on research performed by Chen et al. [21]. The script involves the choice of the resolution of the generated image and the physical dimensions of the area to be covered in RSP. The RSP is built from three sizes of speckles placed randomly around the nodes of a square grid covering the structure. For cooperation with the FEM framework shown in Section 3, a pattern of the size of 4 mm × 400 mm was generated using 0.4 mm as the base circle radius. Its fragment is presented in Figure 2B.

### 2.2. Numerical Setup for Synthetic Image Generation

**Figure 3:**
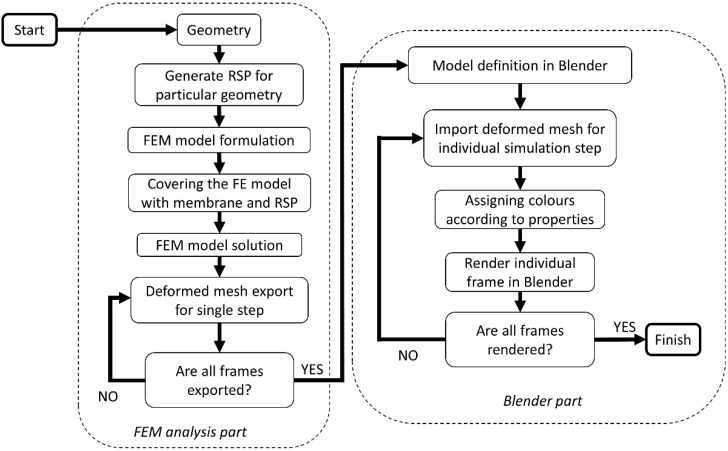
The scheme of the proposed simulation algorithm for video sequence generation with RSP.

The currently presented version of the proposed numerical setup aims to allow for dynamic phenomena simulation such as vibrations of the mechanical structures. Compared to the previously presented computational scheme, this is a significant development. The general principle of simulation is unchanged. The diagram of the developed version of the simulation methodology is shown in Figure 3.

The FE model was formulated based on the geometrical model of the mechanical structure. *Altair* HyperMesh version 2017.1 software was used as a FEM preprocessor. The model was required to be developed from solid (3D) elements. This requirement is mandatory due to later use of the FE mesh as a 3D object in the rendered scene. In the case of structures that could be modeled with 1D or 2D FEs, the necessity to use 3D elements resulted in an increased computational time due to the increased number of degrees of freedom of the model. Nonetheless, using 3D FEs, further processing was necessary in the Blender graphics program. Moreover, a model should be coated with shell elements to create a membrane. This step was necessary to enable the visualization of surfaces in the Blender graphics program. Membrane thickness and material were selected so as not to change the stiffness of the simulated object in a significant way. It is worth noting that the membrane nodes were consistent with the 3D FEs’ nodes, so the compatibility of displacements was ensured. If an RSP is modeled for a given surface of the model, the FEs of the membrane should enable the pixel reproduction of the required pattern. This process was automated using a Matlab script. The corresponding code commands created nodes and elements based on the coordinates of the white and black fields of the designed RSP. In addition, each FE was assigned a property that describes its white or black area membership. 

**Figure 4:**
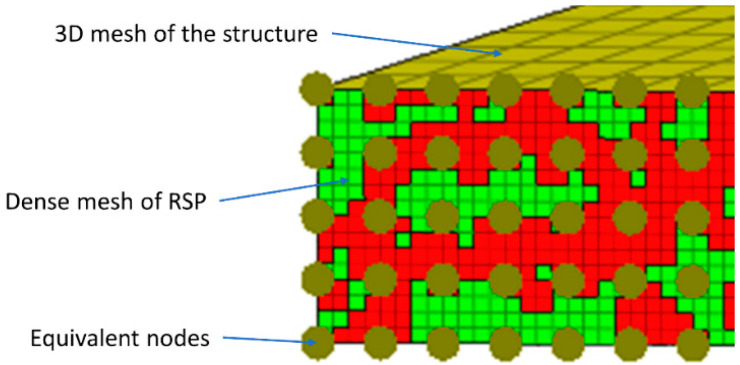
Graphical representation of RSP FE mesh and solid mesh of beam’s structure.

Typically, the size of the RSP fields was smaller than the size of the elements used in the FE model. However, in order to correctly reproduce the RSP, it was necessary to use a very dense grid that allows the reproduction of this pattern. The simplest approach would be to increase the density of the structure’s mesh to such an extent as to achieve sufficiently small elements in the RSP. Nevertheless, such an approach would be very computationally inefficient. In our setup, we proposed importing a separate mesh of RSPs into the existing FEM model of the examined structure and fix together selected nodes of both meshes to make them equivalent. In the case of meshes with regular dimensions, every nth node will be shared by the RSP mesh and the structure, as shown in Figure 4. In the example shown, every 4th node of the RSP mesh is shared by the mesh of the main structure.

This approach ensures the deformation of the RSP together with the main model structure, which is the main goal of the developed methodology. The other necessary parameters of the FE model, such as material properties and boundary conditions, are subsequently defined. The FE model was solved in the next step by employing the *MSC.Marc* solver.

The deformed FE mesh was exported into the *.STL files after completion of the simulation. This is a geometry file that can be loaded into the Blender environment. The export process must be performed for each computational step separately. In addition, a separate file was generated for areas of the simulated structure where a different texture or color was used, which will be defined later. The authors developed a *Python* script to automate this process, which was necessary to automate this procedure for many computational steps. It uses the *py_mentat* and *py_post* libraries, which are available as part of the *MSC.Marc* package. The purpose of the code was to automate the export process of the deformed FE mesh to a large number of separate *.STL files that are needed at a later stage of the algorithm’s operation.

The size of the computational step in the classic dynamic transient simulation is dictated by the criterion of correct representation of the highest frequencies in the model and the convergence criteria of the numerical solution. If the results are further used for the production of synthetic images, there is an additional criterion for the calculation step size: the step size must be a multiple of the frames-per-second (FPS) parameter to enable the correct mapping of time moments in the video sequence.

The next stage of simulation was carried out in the *Blender* software. Its purpose is to generate a synthetic image of a simulated object using computer resources, the so-called *render*. The first stage of the algorithm requires defining the base model in the *Blender* environment. It is needed to determine cameras (position, orientation, sensor size, lens focal length), lighting points (location, intensity), rendering parameters (resolution, output file format, compression), background, and other textures that will later be applied to the imported mesh. In the next step of the algorithm, the deformed FE mesh fragments were imported into the *Blender* software. This is done for each frame of the video sequence. It is important that the areas where separate textures need to be defined are imported as separate files. This approach allows the application of different textures to these areas in a graphics program. Then, the scene was rendered using the *Blender* graphics engine. As a result, a digital image of the simulated scene was created synthetically. Rendering of individual scenes was performed for all required frames of the video sequence. This repetitive stage was also automated by a *Python* script. The last step was to assemble the rendered images into a synthetic video sequence using the *Video Sequencer* module. 

## 3. Case Study—Cantilever Beam

A case study is provided and discussed to illustrate the method of conducting a dynamic simulation of a structure with an RSP. The simulated sample is a cantilever beam with the following dimensions: 800 × 40 × 4 mm. It is made of an aluminum alloy. The FEM plus Blender simulation aims to generate a video sequence of imposed vibrations at natural frequencies of the beam with the RSP attached to it.

### 3.1. FEM Model: Beam with RSP

According to the algorithm described in Section 3, in the first step, a geometric model of the studied structure is defined. Then, the geometry is meshed with 3D (solid) FEs. In the next stage, the properties of the FEs are described. The model adopts the material parameters given in Table 1.

**Figure 5:**
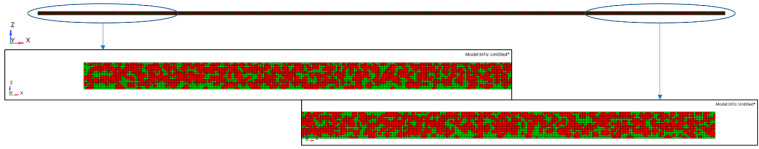
Graphical representation of RSP FE mesh in *Altair Hyper Mesh* pre-processor.

The next stage is to define the RSP on the side surface of the sample. The RSP was prepared with a resolution four times higher than the dimensions of the surface on which it is modeled, i.e., 3200 × 16 fields of black or white color. Data structures describing the positions of white and black fields of the RSP on the side surface of the modeled sample were created as a result of designing the RSP stage. A *Matlab* script was developed to allow automatic generation of the FE model file (saved in the *.bdf format) with elements representing white and black speckle areas. In total, the number of elements representing the RSP is 51,200 (16 × 3200 fields). A graphical representation of the generated FE mesh is shown in Figure 5.

Red and green colors were used to visualize the assignment of FE mesh elements into two types of properties. At a later stage of applying textures in Blender, this division will be used to assign white or black colors to these areas. In the next step, the prepared FE mesh of the RSP model was imported into the primary FE model of the beam.

The dimensions of the imported RSP mesh and the side surface of the beam model were consistent. The size of the FEs of RSP was smaller than the size of the FEs of the primary beam model. The overlapping nodes of two FE meshes were made equivalent (fixed together). In the analyzed case, every 4th node of the RSP model was common with the beam’s nodes. The membrane on the remaining areas of the beam (2D FEs) was also defined on the remaining surfaces of the beam. These shell-type elements form a closed volume, which was imported into the Blender program. The thickness of membrane elements was set to 0.01 mm to minimize their impact on the stiffness of the model.

### 3.2. Influence of Membrane FEs on Dynamic Properties of the Simulated Structure

**Figure 6:**
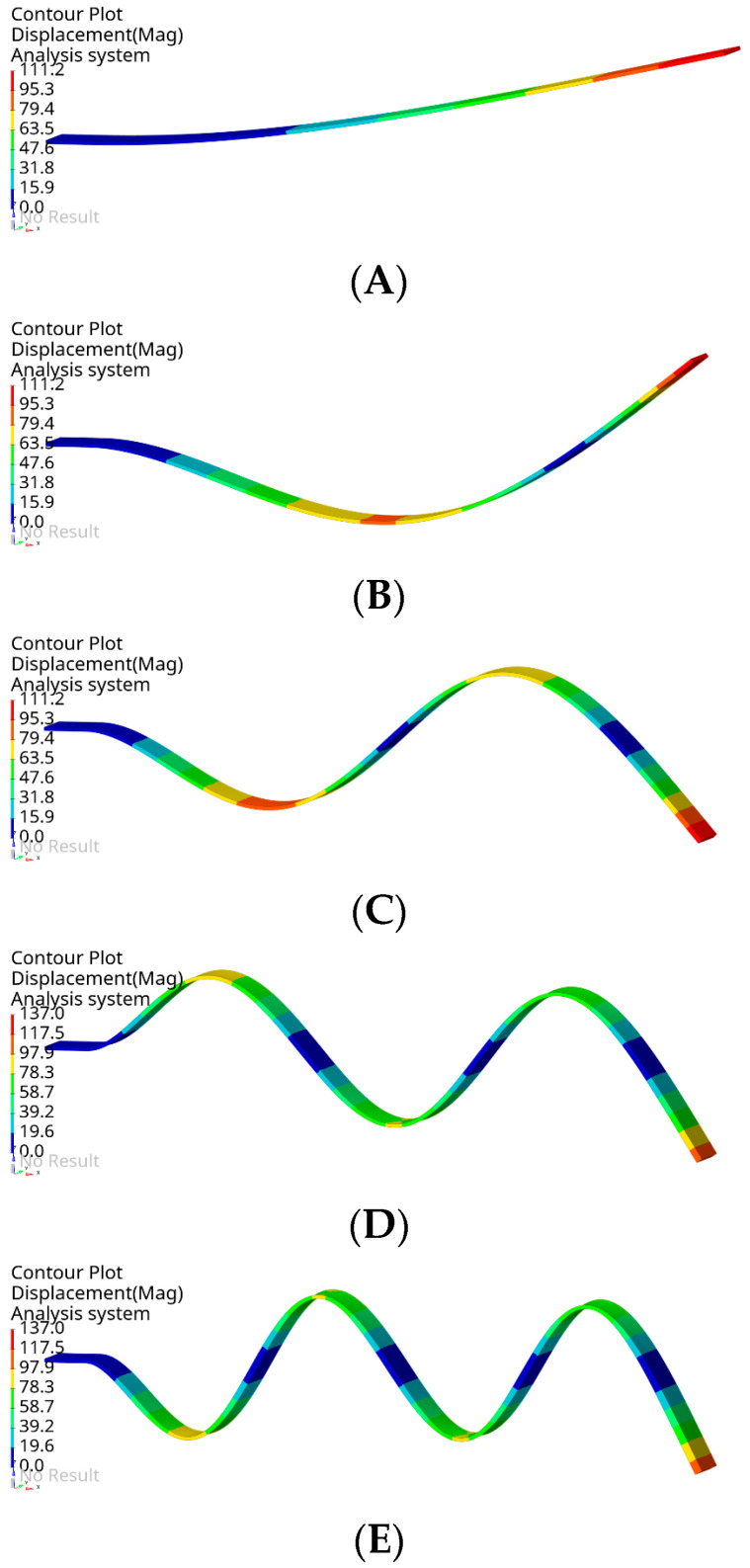
Graphical representation of computed normal modes: first bending mode (**A**), second bending mode (**B**), third bending mode (**C**), fourth bending mode (**D**), and fifth bending mode (**E**).

Covering the model with 2D elements (membranes) was required to allow saving the mesh into the *.STL format, which can be imported into the Blender program. Modal analysis of the beam with and without membrane elements was carried out as a comparative study to determine the influence of the membrane elements on the dynamic properties of the model. The results are presented in this section. Moreover, experimental results were also obtained. The visualization of the considered beam’s modes is illustrated in Figure 6.

The analysis was limited to the first five bending modes. The obtained results are presented in Table 2.

The results allow us to summarize that the use of membrane FEs affects the stiffness of the entire structure to a negligible degree. The differences in the determined natural frequencies for the two numerical models are below 0.5%. In addition, the difference between the experimental and numerical results obtained using the model with membrane FE is in the range of 0.3–5.0%. The highest difference was determined for mode #2. Therefore, it can be concluded that using additional elements (2D) required for further processing of the model (in Blender) does not affect the structure’s stiffness. The second conclusion is that the formulated numerical model sufficiently reflects the dynamics of the actual object.

### 3.3. Dynamic Transient Simulation

**Figure 7:**
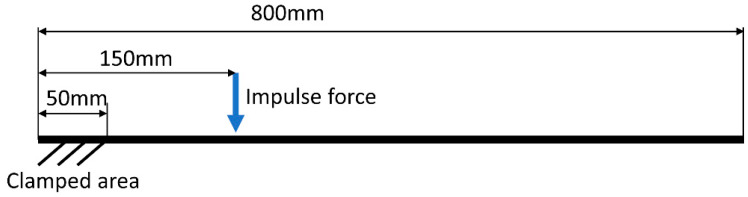
Scheme boundary conditions applied in dynamic simulation.

As written in the introduction in Section 4, the output presented in this example should consist of a sequence of synthetically generated images depicting the excited vibrations of a cantilever beam. To this end, it is necessary to render images for subsequent moments and combine them into a video sequence. Therefore, it is necessary to determine the displacements of the nodal points of the model at successive moments. The defined computational case is of the dynamic transient type. Boundary conditions consider loading the sample by impulse force and one-sided fixation of the beam. An impulse force has an amplitude of 100 N and lasts for 0.002 s. The beam boundary conditions scheme is presented in Figure 7.

The Dynamic Transient Modal Superposition algorithm was used to obtain the structure’s response to the impulse excitation by taking into account only selected modes of natural vibrations. This method for solving dynamic problems is available in the *MSC.Marc* solver [22]. Responses at resonant frequencies of 106.3 Hz, 208.4 Hz, and 344.5 Hz were obtained. The time step size in the dynamic transient simulation was set to 0.1 ms. The requirement to obtain at least 20× higher sampling than the highest analyzed natural frequency (344.5 Hz) dictated the choice of the computational step size. The step size also corresponds to the FPS parameter (10,000 FPS) of rendered video sequence, as introduced in Section 3. The simulation time was set to 0.1 s. Considering the utilized time step (0.1 ms) and simulation time, the output results were computed for 1000 steps. The simulation parameters adopted were the same for all three analyzed cases and allow for appropriate sampling for the highest simulated frequencies (344.5 Hz) and simultaneous simulation of a minimum of 10 vibration periods for the lowest stimulated mode (106.3 Hz). According to the algorithm described in Section 2.2., the deformed mesh is exported next into *.*STL* files for each increment. These files are the input data for further processing in the Blender graphics program.

### 3.4. Blender Setup

**Figure 8:**
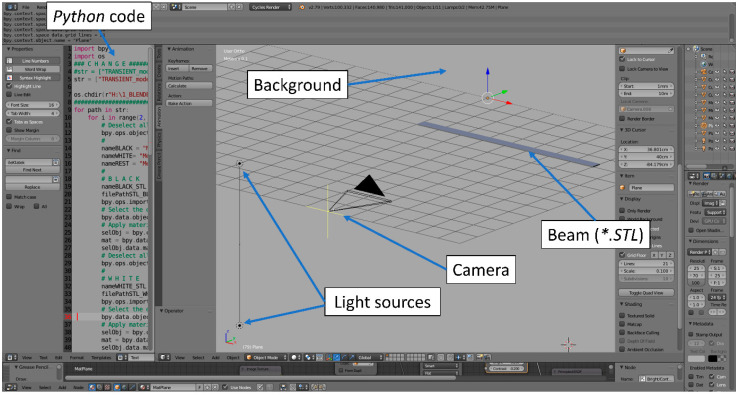
The model in the Blender environment for the beam’s deflection rendering process.

The most important settings of the modeled vision system were set first: the position of the camera relative to the beam, lighting points, and the background textures. Textures applied to the selected fragments of the beam were also defined. The high-speed camera model Phantom VEO-E 340L, which is characterized by a 26 × 16 mm sensor size and a lens with a focal length of 30 mm, was considered in the simulation setup. In the analyzed case, the simulated camera was centrally located in front of the beam and is 1 m away. The camera allows for capturing images with a high resolution of 2560 × 800 pixels. The field of view of the camera was limited to 2560 × 70 pixels to exclude irrelevant background areas. This allows a reduction in the size of images and a decrease in rendering and further processing time. Two-point light sources were located 500 mm behind the camera, below and above (±300 mm) the sample level. The view of the formulated model in the Blender program is shown in Figure 8.

**Figure 9:**
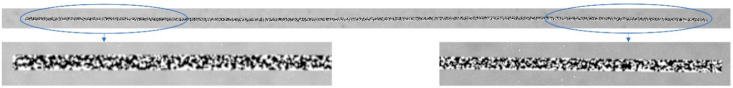
Exemplary rendered synthetic image presenting the side surface of the beam with RSP.

The developed Python script allows automation of the process of importing the deformed structure’s mesh and rendering of the scene. As a result, a synthetic image of a side view of the beam was generated. On the side surface of the beam, the RSP was modeled as discussed earlier. An example of a synthetically generated frame is shown in Figure 9. The RSP on the side surface of the beam is clearly visible, especially in the magnified view of both ends. After the script was completed and 1000 frames were generated according to animation time (t = 0.1 s at 10,000 FPS), the sequence of frames was assembled into an animation and saved as a *.avi file. Synthetically generated vision data of dynamic phenomena were then ready for further processing.

The DIC method was implemented in the *Matlab* programming environment to compute the beam’s deformation in each of the beam’s vibrational modes. The parameters of the algorithm were chosen as follows: the size of a single DIC template was 12 × 12 pixels, the distance between each adjacent template was 12 pixels (there was no overlap between templates), and the search window’s size was 60 × 60 pixels. The number of measurement points was 200. The scale coefficient was computed based on the metric dimensions of the FE model independently in the X and Y directions. The value of the scale coefficient amounted to 0.333 mm/pix and 0.307 mm/pix for the X and Y directions, respectively.

## 4. Results and Discussion

**Figure 10:**
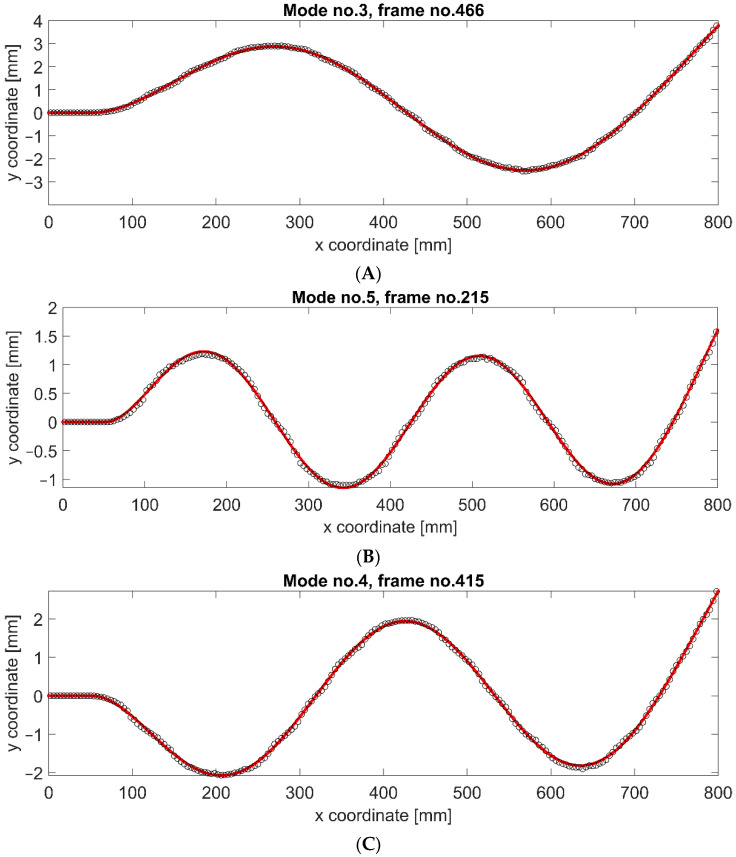
Mode shapes of cantilever beam computed using synthetic images with RSP employing DIC processing technique (circles), and numerical results from FEA (red line): mode no. 3 (**A**), mode no. 4 (**B**), and mode no. 5 (**C**).

The use of DIC of RSP made it possible to determine the displacements of individual beam sections for all tested cases. The analysis was performed for all the synthetic frames of the generated video sequences. Then, we compared the obtained displacement results with the original FEA results. This comparison shows whether the proposed RSP modeling approach and synthetic image generation algorithm for dynamically loaded structures introduce significant distortions. Figure 10 shows three modes of vibration at the moment of maximum deflection.

**Figure 11:**
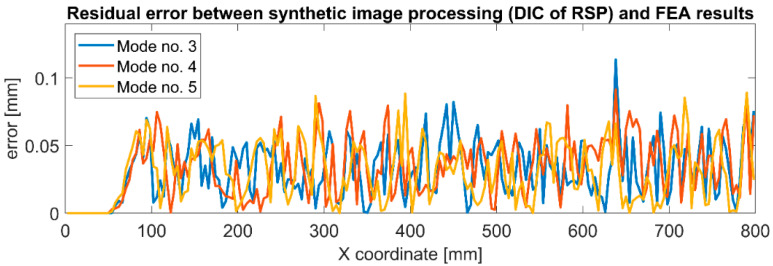
Residual error between synthetically generated images with RSP employing DIC technique and FEA results for considered bending modes of the cantilever beam.

The solid (red) line shows the results directly from the FEA simulation, while the circle-shaped markers represent the results of synthetic image processing using DIC. The results of DIC of RSP processing are highly consistent with the original FEA data. There are no significant differences in individual mode shapes, although they differ significantly in vibration amplitudes. A residual error was determined to quantify the differences between both datasets. The results are presented in Figure 11.

It can be seen that in the constrained area, the error is 0 mm. Then, for the X coordinate in the range of 500–800 mm, this error increases for all vibration modes. In this fragment, which is very close to the constrained area, the displacements are small in all cases. In the rest of the structure, the error is similar along the entire remaining length and is not more significant than the error occurring close to the constrained area. The statistical parameters describing the residual error for the individual mode shapes are presented in Table 3.

**Figure 12:**
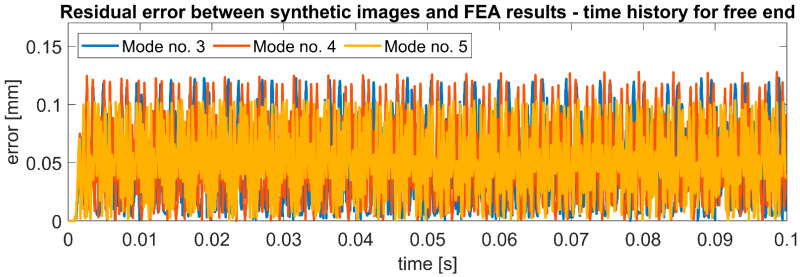
Residual error between synthetically generated images with RSP employing DIC technique and FEA results for considered bending modes of cantilever beam—vertical position time history for the free end.

It can be observed that the maximum error increases for the modes characterized by higher vibration amplitudes, but the differences are minor: 0.025 mm with an amplitude difference of 2.21 mm. However, the average error and STD are at a similar level for all mode shapes. These observations prove that the error is not introduced by the method of RSP modeling nor the technique of synthetic video frame generation. It is most likely related to sub-pixel interpolation, especially considering the scale factor of 0.307 mm/pix and the maximum error of 0.114 mm, which is almost three times less than the length of one pixel in the image. Figure 12 shows the residual error for the position of the free end of the beam in time history. It can be observed that the error is repeatable for successive moments of time and remains at the same level.

It can therefore be concluded that the deformable RSP modeling approach is correct, and the noted errors are related to the sub-pixel interpolation of the DIC algorithms. Thus, an efficient RSP modeling method for synthetic vision data has been successfully validated for an adopted pseudo-experimental setup. The proposed results were obtained for one particular laboratory setup. Therefore, despite the fact that the results are promising, further evaluations are still required, especially if the method is to be applied for complex-geometry structures. Similar to the design of FE models, the approach requires validation in terms of the rendering setup of Blender software to provide good overlap with experimental results, but this should be investigated on a case-by-case basis depending on the particular structure and vision system at hand.

## 5. Summary and Concluding Remarks

As part of this work, a simulation environment for generating synthetic video data with deformable RSP was developed. The proposed approach used the FEA results to determine the simulated model’s deformed shape. Next, the Blender environment was used to render a scene of the structure under study, whose deformation was provided by the FEA results. An approach to modeling RSP, which deforms together with the system, is a significant contribution presented in this paper. RSP representation is often used in practice to capture small deformations of the observed structures correctly. This feature was successfully implemented and tested in the simulation environment developed within this work. The simulation scheme was validated with a case study of excited vibrations of a cantilever beam with RSP applied on its surface. Nonetheless, the method presented in this paper is universal and is not limited to beam-like structures. Other structures will only require the development of an appropriate FE mesh representing the RSP. It is also possible for static simulations to be performed, or different types of RSPs may be applied. As shown in Section 4, synthetic images accurately reflect the structure’s deformations since the residual error between displacements computed based on synthetic images with RSP employing the DIC technique and pure FEA results is almost three times smaller than the size of the pixel in the image. However, this quantitative comparison may be applied only to the examined case presented in this paper. This error will depend on the modeled focal length of the lens, image resolution, and the distance from the simulated construction; it is possible to obtain smaller or greater values of this error. Nevertheless, the presented case study summarizes that the proposed methodology of generating synthetic video data for structures with deformable RSP is adequate. The observed errors in determining displacements are related to the sub-pixel interpolation used in the DIC algorithm.

Testing various conditions of the vision system models is one of the directions of our future work. The proposed method can be used for simulating damaged structures as long as the damage can be correctly modeled using the FEM models based on 3D elements. Material defects can be modeled, for example, as local changes in stiffness, mass, or damping in the model. Cracks (in a simplified linear approach) can be simulated as disconnected nodes in the FEM model. The second direction of our future work will be simulations of damaged test samples accompanied by experiments for model validation.

## Figures and Tables

**Table 1:** Material properties used in the FE model.

Density	2700 kg/m^3^
Young’s Modulus	69.5 GPa
Poisson ratio	0.33

**Table 2:** Results of natural frequency comparison.

	Natural Frequency [Hz]			Relative Error	
Bending Mode Number	Model without Membrane FE	Model with Membrane FE	Experiment	Model without vs. with Membrane FE	Model with Membrane FE vs. Experiment
#1	6.03	6.06	---	0.46%	---
#2	37.8	38.0	40.0	0.45%	5.0%
#3	105.8	106.3	110.0	0.47%	3.4%
#4	207.4	208.4	209.0	0.48%	0.3%
#5	342.9	344.5	347.0	0.46%	0.7%

**Table 3:** Statistical parameters of residual error between synthetic images processing and FEA results for different mode shapes.

	The Amplitude at the Free End [mm]	Residual Error		
		Max [mm]	Mean [mm]	STD [mm]
Mode no. 3	3.78	0.114	0.032	0.022
Mode no. 4	2.72	0.091	0.034	0.022
Mode no. 5	1.57	0.089	0.030	0.022

## Data Availability

Data used in this article are available on demand from the corresponding author.
